# Physicochemical and Biological Modifications in Mesenchymal Stem Cells-Derived Conditioned Media Under Hypoxic Preconditioning: Impact on Oxidative Stress and Nanoparticle Stability

**DOI:** 10.3390/life15050702

**Published:** 2025-04-26

**Authors:** Gülsemin Çiçek, Fatma Öz Bağcı, Mehmet Filizfidan, Selçuk Duman, Tahsin Murad Aktan

**Affiliations:** 1 Department of Histology and Embryology, Faculty of Medicine, Necmettin Erbakan University, Konya 42090, Türkiye; gulseminyuksel@gmail.com (G.Ç.); drfatmaozbagci@gmail.com (F.Ö.B.); dumanselcuk@gmail.com (S.D.); 2 Graduate School of Health Sciences, Interdisciplinary Department of Stem Cells and Regenerative Medicine, Ankara University, Ankara 06520, Türkiye; mfilizfidan@selcuk.edu.tr; 3 Advanced Technology and Research Center, Selçuk University, Konya 42130, Türkiye

**Keywords:** mesenchymal stem cells, hypoxic preconditioning, oxidative stress, conditioned media, nanoparticle stability, HIF-1α

## Abstract

Hypoxic preconditioning (HP) is a promising approach to enhance the therapeutic efficacy of mesenchymal stem cells (MSCs) by modulating their oxidative stress response, metabolic activity, and secretome composition. Conditioned media (CM) obtained from MSCs cultured under hypoxia contains bioactive molecules and extracellular vesicles (EVs) that support regenerative processes. However, the effects of varying oxygen levels on the redox status and physicochemical characteristics of MSC-derived CM remain incompletely understood. This study aimed to investigate how two physiologically relevant oxygen concentrations (1% and 5%) influence oxidative stress parameters and nanoparticle features in Wharton’s jelly-derived MSC (WJ-MSC)-conditioned media. Cells were cultured under 1% or 5% O_2_ and subjected to serum starvation for 48 or 72 h. CM samples were analyzed for total oxidant status (TOS), total antioxidant status (TAS), and oxidative stress index (OSI). Nanoparticle size and zeta potential were evaluated using dynamic light scattering (DLS), and HIF-1α levels were quantified via ELISA. Results showed that CM from 1% O_2_ cultures exhibited significantly higher oxidative stress, with elevated TOS and OSI values and reduced TAS levels, particularly after 72 h. Nanoparticle size was initially larger under 1% O_2_ but decreased with time, whereas 5% O_2_ supported more stable size profiles. Zeta potential measurements revealed more negative values under 5% O_2_, indicating greater colloidal stability. HIF-1α expression markedly increased under 1% O_2_, confirming hypoxia-induced cellular adaptation. In conclusion, this study demonstrates that graded hypoxia distinctly modulates oxidative stress and nanoparticle characteristics in MSC-derived CM. These findings provide a basis for optimizing hypoxic preconditioning protocols to improve the quality and therapeutic potential of acellular MSC-based therapies.

## 1. Introduction

Mesenchymal stem cells (MSCs) derived from Wharton’s jelly (WJ-MSCs) are widely recognized for their regenerative potential, immunomodulatory properties, and paracrine effects [1]. The microenvironment, particularly oxygen (O_2_) levels, plays a critical role in regulating MSC behavior, including their proliferation, differentiation, and secretome composition. Physiological oxygen levels in tissues range between 1 and 7%, with lower oxygen levels (hypoxia) influencing the metabolic activity and survival mechanisms of MSCs. Hypoxic preconditioning, achieved by culturing MSCs under reduced O_2_ levels, has been shown to enhance their therapeutic potential by altering gene expression patterns, increasing hypoxia-inducible factor-1 alpha (HIF-1α) activation, and modulating the secretion of bioactive molecules [2].

O_2_ concentration is a critical factor influencing mesenchymal stem cell (MSC) behavior; it affects the proliferation, differentiation, and secretion of bioactive molecules. In vitro studies commonly utilize normoxia (≈21% O_2_), but physiological O_2_ levels in various tissues range between 1 and 7%, with stem cell niches often existing in hypoxic environments [3]. Culturing MSCs under hypoxia (≤5% O_2_) has been shown to enhance their survival, multipotency, and paracrine effects [4]. Exposure to 1% oxygen has been shown to stabilize hypoxia-inducible factor-1 alpha (HIF-1α), which leads to increased expression of angiogenic factors such as vascular endothelial growth factor (VEGF) and a metabolic shift favoring glycolysis over oxidative phosphorylation [5,6]. For example, studies have demonstrated that culturing Wharton’s jelly-derived mesenchymal stem cells (WJ-MSCs) under 1% oxygen conditions enhances their therapeutic properties in ischemic models. Additionally, culturing MSCs under 5% oxygen conditions has been associated with increased extracellular vesicle production, thereby improving regenerative capacity [7,8]. Understanding these oxygen-dependent mechanisms is essential for optimizing MSC culture conditions for therapeutic applications.

Conditioned media (CM) derived from MSC cultures contains a rich composition of extracellular vesicles (EVs), cytokines, and growth factors, making it an attractive acellular alternative for regenerative medicine [9]. Recent clinical investigations suggest that MSC-derived CM exhibits anti-inflammatory, immunomodulatory, and pro-angiogenic effects, which contribute to tissue repair without the risks associated with cell transplantation [10]. Several studies have demonstrated the therapeutic potential of mesenchymal stem cell-derived conditioned medium (MSC-CM) in treating various conditions. For instance, one study found that bone marrow-derived MSC-CM significantly improved wound healing in diabetic foot ulcers in rats by accelerating wound closure, promoting cell proliferation, and enhancing angiogenesis [11]. Another study reported that intra-articular injections of MSCs effectively attenuated osteoarthritis progression, as evidenced by improved joint space width and articular cartilage surface continuity [12]. These findings suggest that MSC-CM holds promise as a therapeutic agent in regenerative medicine. Moreover, CM-derived exosome therapy is being explored in clinical trials for neurological disorders, cardiovascular diseases, and autoimmune conditions [13,14,15]. Advances in CM standardization and large-scale production are crucial to translating these findings into clinical practice.

One of the key mechanisms underlying hypoxic preconditioning is the activation of hypoxia-inducible factor-1 alpha (HIF-1α), a transcription factor that regulates cellular responses to low O_2_ levels. HIF-1α stabilization in hypoxic MSCs promotes angiogenesis, metabolic reprogramming (switch to glycolysis), and anti-apoptotic pathways, ultimately enhancing cell viability and therapeutic effectiveness [2,16]. Studies have shown that preconditioned MSCs exhibit increased secretion of vascular endothelial growth factor (VEGF), hepatocyte growth factor (HGF), and stromal-derived factor-1 (SDF-1), which contribute to tissue repair and regeneration [17,18]. Hypoxic preconditioning has been shown to enhance the secretion of extracellular vesicles (EVs), improve exosome cargo composition, and strengthen paracrine signaling, thereby offering a promising approach for cell-free therapies. For instance, one study demonstrated that mesenchymal stem cell (MSC)-derived exosomes from hypoxic cultures exhibited superior neuroprotective effects compared to those from normoxic conditions [19]. Additionally, preconditioned MSCs have shown improved outcomes in preclinical models of ischemic stroke, myocardial infarction, and osteoarthritis [20,21].

Nanoparticle size and surface charge (zeta potential) are critical parameters that influence the stability, uptake, and therapeutic potential of extracellular vesicles in regenerative medicine applications [22,23]. Previous studies have indicated that hypoxia enhances the production of smaller, bioactive vesicles enriched with growth factors, microRNAs, and anti-inflammatory molecules [24]. However, it remains unclear how different levels of hypoxic exposure influence the physicochemical properties of secreted nanoparticles and whether extended starvation periods further modulate their characteristics [22].

This study aims to provide insights into how different hypoxic preconditioning conditions impact the nanoparticle composition of MSC-derived conditioned media, which is essential for optimizing extracellular vesicle-based therapies. By comparing the size, surface charge, and protein content of secreted nanoparticles, we aim to determine the optimal preconditioning strategy for generating MSC-derived CM with enhanced therapeutic efficacy. Unlike previous studies that have examined isolated parameters such as oxidative stress or vesicle size under hypoxia, our work uniquely integrates redox biomarkers (TAS, TOS, OSI), dynamic nanoparticle characteristics (DLS, zeta potential, electrophoretic mobility), and HIF-1α-mediated signaling within the same experimental design. Moreover, by focusing on two physiologically relevant oxygen tensions (1% and 5% O_2_) rather than including a supraphysiological normoxic condition, this study offers a more contextually relevant and mechanistic perspective on how graded hypoxia modulates the MSC secretome. To our knowledge, this is the first study to systematically correlate oxygen-dependent redox imbalance with nanoparticle-level alterations and hypoxia-inducible signaling in MSC-conditioned media. These findings offer a novel experimental platform for optimizing secretome-based strategies in regenerative medicine.

## 2. Materials and Methods

### 2.1. Cell Culture and Hypoxic Preconditioning

#### 2.1.1. Isolation and Expansion of Wharton’s Jelly-Derived Mesenchymal Stem Cells (WJ-MSCs)

Wharton’s jelly-derived mesenchymal stem cells (WJ-MSCs) were obtained from (PCS-500-010, Manassas, VA, USA) and cultured in Dulbecco’s Modified Eagle Medium (DMEM) (Gibco, Grand Island, NY, USA) supplemented with 10% fetal bovine serum (FBS) (Gibco, Grand Island, NY, USA), 1% penicillin–streptomycin, and 1% L-glutamine (Gibco, Grand Island, NY, USA) at 37 °C with 5% CO_2_ under normoxic conditions (21% O_2_). Cells were passaged at 70–80% confluency using 0.25% trypsin-EDTA solution (TrypLE Express Enzyme, Gibco, Grand Island, NY, USA) and used for experiments between passages 2 and 4.

#### 2.1.2. Hypoxic Preconditioning and Conditioned Medium Collection

For hypoxic preconditioning, WJ-MSCs were seeded in culture plates and maintained in a hypoxia chamber (Stemcell Technologies Inc., Vancouver, BC, Canada) under controlled O_2_ conditions of either 5% or 1% O_2_. The chamber was continuously monitored for O_2_ concentration, temperature, and humidity to ensure stable conditions. Following preconditioning, cells were further processed for metabolic activity assays and conditioned media collection.

### 2.2. Cell Lysate Preparation

To evaluate intracellular antioxidant levels, cell lysates were prepared from mesenchymal stem cells (MSCs) cultured under different oxygen concentrations (5% and 1%). The following protocol was applied for lysate preparation:

At the designated time points (48 and 72 h), culture media was aspirated, and adherent cells were washed twice with ice-cold PBS (phosphate-buffered saline, pH 7.4) to remove residual conditioned medium and extracellular contaminants. The PBS was completely aspirated before proceeding to lysis. Cells were detached using a cell scraper on ice to avoid mechanical stress-induced degradation of proteins. The cell pellet was resuspended in ice-cold RIPA buffer (Radioimmunoprecipitation Assay Buffer) containing the following: 50 mM Tris-HCl (pH 7.4), 150 mM NaCl, 1% Triton X-100, 0.5% Sodium deoxycholate, and 0.1% SDS (Sodium Dodecyl Sulfate). The samples were incubated on ice for 30 min, with gentle vortexing every 5 min to facilitate lysis. The lysate was centrifuged at 2000× *g* for 15 min at 4 °C to separate the soluble protein fraction from cell debris and unbroken organelles. The supernatant (containing total soluble proteins) was transferred to a pre-chilled microcentrifuge tube and stored at −80 °C for further biochemical analyses.

### 2.3. Metabolic Activity and Mitochondrial Analysis

#### 2.3.1. MTT Assay for Cell Viability

The metabolic activity of WJ-MSCs was assessed using the MTT assay (CyQUANT™ MTT Cell Viability Assay Kit, Invitrogen, Thermo Fisher Scientific Inc., Waltham, MA, USA) at 48, 72, and 96 h. Cells were seeded in 96-well plates and incubated with MTT reagent (5 mg/mL) for 4 h at 37 °C. The resulting formazan crystals were dissolved in DMSO, and absorbance was measured at 570 nm using a microplate reader (Allsheng, AMR-100, Hangzhou Allsheng Instruments Co., Ltd., Hangzhou, China)

#### 2.3.2. MitoTracker Staining for Mitochondrial Activity

Mitochondrial activity was assessed using MitoTracker Green (Invitrogen, Carlsbad, CA, USA). Cells were incubated with 200 µM MitoTracker dye for 30 min at 37 °C, followed by PBS washing. Fluorescence imaging and confocal microscopy analyses were performed using the Nikon A1R+ confocal microscope (Nikon Corporation, Tokyo, Japan). The system is equipped with a high-speed resonant scanner and a galvano scanner for high-resolution imaging. Mitochondria in cells were stained with MitoTracker Green, nuclei were stained with DAPI (blue), and fluorescence images were captured at different magnifications (10× and 40×). For fluorescence detection, the excitation and emission wavelengths were set as follows: MitoTracker Green: Excitation at 488 nm, emission at 500–550 nm. DAPI: Excitation at 405 nm, emission at 450 nm. Fluorescence intensity was quantified using ImageJ software version 1.53 (National Institutes of Health, Bethesda, MD, USA). The analysis included background subtraction, mean intensity measurements, and normalization to ensure accurate comparisons between experimental groups. Data were expressed as mean fluorescence intensity per cell.

### 2.4. Hypoxia-Related Protein Analysis ELISA for HIF-1α Expression

Cell lysates were collected at 48, 72, and 96 h, and HIF-1α levels were quantified using a commercial human HIF-1α ELISA kit (Sunred Biotechnology, Shanghai, China). Absorbance was measured at 450 nm, and results were normalized to total protein concentrations.

### 2.5. Conditioned Media Collection and Nanoparticle Analysis

#### 2.5.1. Starvation and Conditioned Media Collection

After hypoxic preconditioning, cells were washed with PBS and incubated in serum-free DMEM for either 36 or 48 h. Conditioned media (CM) was collected, centrifuged at 300× *g* for 10 min to remove debris, and stored at −80 °C.

#### 2.5.2. Nanoparticle Size and Zeta Potential Analysis

The size distribution and zeta potential of nanoparticles in the conditioned media were analyzed using dynamic light scattering (DLS)**.** No additional purification or concentration procedures were applied to the conditioned media before nanoparticle size and zeta potential analysis in order to preserve the native characteristics of the secretome. Measurements were performed using the Malvern Zetasizer Nano ZS (Malvern Panalytical, Malvern, UK) equipped with the MPT-2 autotitrator module. For size distribution analysis, samples were diluted in filtered distilled water to prevent aggregation and analyzed at 25 °C using a 173° backscatter detection setup. For zeta potential measurements, electrophoretic mobility was determined via phase analysis light scattering (PALS). Measurements were taken at room temperature under standard conditions. All DLS experiments were conducted in triplicate, and results were reported as mean ± standard deviation (SD).

#### 2.5.3. Total Protein Quantification

The Bradford assay kit (ABPbio, Beltsville, MD, USA, Cat. No. P011) was used to determine exosome concentration. The Bradford Protein Assay Kit is a colorimetric assay designed for the rapid and simple quantification of protein concentrations. Protein concentrations were calculated based on a standard curve generated using serial dilutions of a protein standard. The assay was performed using a 96-well ELISA plate. Each experimental group was analyzed in triplicate, and the results were averaged.

First, a series of twofold serial dilutions of Bovine Serum Albumin (BSA) standards were prepared within the range of 2000 µg/mL to 0 µg/mL. A volume of 10 µL from each standard and unknown sample was pipetted into the appropriate microplate wells. For the blank control, PBS was added. Subsequently, 200 µL of Bradford reagent was added to each well, and the plate was mixed for 1 min using a plate shaker. The plate was then incubated at room temperature for 5 min. Absorbance measurements were taken at 595 nm.

The blank-corrected average absorbance values at 595 nm for each BSA standard were plotted against their respective protein concentrations (µg/mL) to generate a standard curve in Excel. This standard curve was used to determine the protein concentration of each unknown sample.

### 2.6. Oxidative Stress Analysis

#### 2.6.1. Total Oxidant Status (TOS) Measurement

The Total Oxidant Capacity (TOS) was quantified using a commercially available colorimetric assay kit (Rel Assay Diagnostics, Gaziantep, Türkiye). The method is based on the oxidation of ferrous ion (Fe^2+^) to ferric ion (Fe^3+^) in the presence of oxidants in the sample, which is then detected by a chromogenic reagent forming a colored complex. Reagent 1 was added to the samples absorbance was measured (A1) at 530 nm using a microplate reader (Allsheng, AMR-100, Hangzhou Allsheng Instruments Co., Ltd., Hangzhou, China). Reagent 2 was added for stabilization and incubation at 37 °C for 5 min to initiate the oxidation reaction. The absorbance (A2) was measured at 530 nm using a microplate reader. The absorbance was calculated by subtracting A1 from A2.

TOS (μmol H_2_O_2_ Eq/L) = (Sample Absorbance/Standard Absorbance) × Standard Concentration

#### 2.6.2. Total Antioxidant Status (TAS) Measurement

The Total Antioxidant Capacity (TAS) was assessed using a Trolox-equivalent antioxidant assay, which measures the ability of antioxidants in the sample to reduce ABTS radical cation (2,2’-azino-bis(3-ethylbenzothiazoline-6-sulfonic acid)), forming a color change detected spectrophotometrically. Reagent 1 was added to the samples absorbance was measured (A1) at 660 nm using a microplate reader (Allsheng, AMR-100, Hangzhou Allsheng Instruments Co., Ltd., Hangzhou, China). Reagent 2 was added for stabilization and incubation at 37 °C for 5 min to initiate the oxidation reaction. The absorbance (A2) was measured at 660 nm using a microplate reader. The absorbance was calculated by subtracting A1 from A2. The TAS levels were determined using a Trolox standard curve, and results were expressed in mmol Trolox Eq/L.

TAS (mmol Trolox Eq/L) = (Sample Absorbance/Standard Absorbance) × Standard Concentration

#### 2.6.3. Oxidative Stress Index (OSI) Calculation

The Oxidative Stress Index (OSI) was calculated to determine the overall oxidative stress status by taking the ratio of TOS to TAS and multiplying by 100 to adjust for scale differences. A higher OSI value indicates an increased oxidative stress burden, whereas a lower OSI value suggests a better antioxidant defense capacity.

OSI = (TOS/TAS) × 100

### 2.7. Statistical Analysis

All experiments were performed in triplicate (*n* = 3), and data were expressed as mean ± standard deviation (SD). Statistical analysis was conducted using SPSS (version 29.0.0). Differences between groups were assessed using one-way ANOVA followed by Tukey’s post hoc test, with *p*-values < 0.05 considered statistically significant.

## 3. Results

### 3.1. MTT Analysis Results

**Figure 1:**
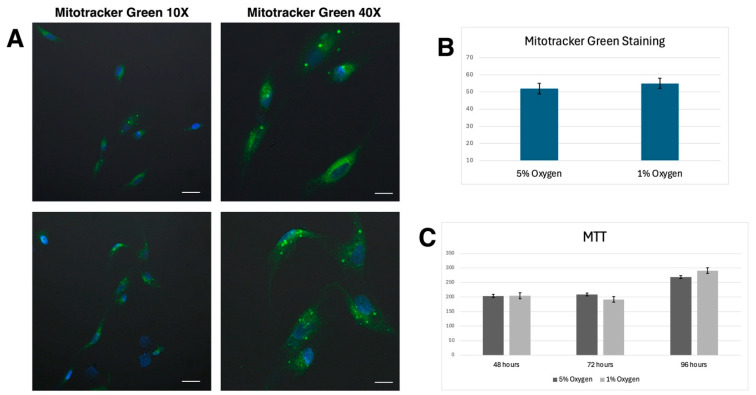
Mitochondrial activity and metabolic assays under different oxygen conditions: (**A**) MitoTracker Green Staining: Representative confocal microscopy images of mesenchymal stem cells (MSCs) cultured under 5% (**top**) and 1% (**bottom**) oxygen conditions. Mitochondria were labeled with MitoTracker Green, while nuclei were counterstained with DAPI (blue). Images were acquired at 10× (**left**) and 40× (**right**) magnifications. Scale bars: 200 µm and 25 µm. (**B**) Quantification of MitoTracker Green Staining: Bar graph comparing the fluorescence intensity of MitoTracker Green-stained MSCs under 5% and 1% oxygen conditions. No statistically significant differences were observed between the two groups, suggesting that hypoxia did not markedly alter mitochondrial content. (**C**) MTT Assay for Metabolic Activity: The metabolic activity of MSCs cultured under 5% and 1% oxygen conditions was assessed using the MTT assay at 48, 72, and 96 h. While no significant differences were observed at 48 h, metabolic activity was transiently reduced at 72 h in the 1% oxygen group. However, at 96 h, cells cultured under hypoxia exhibited a trend toward higher metabolic activity compared to those maintained under 5% oxygen, suggesting an adaptive response to low oxygen conditions.

At 72 h, a statistically significant difference in metabolic activity was observed between the 5% and 1% oxygen groups (*p* = 0.018), whereas no significant differences were detected at 48 and 96 h (*p* > 0.05). At 48 h, the mean MTT value in the 5% oxygen group was 204.00 ± 19.47, while in the 1% oxygen group it was 204.50 ± 23.73. The independent samples t-test showed no significant difference between the groups (*p* = 0.964). At 72 h, the 5% oxygen group exhibited significantly higher metabolic activity (208.50 ± 12.92) compared to the 1% oxygen group (192.00 ± 11.66), with a mean difference of 16.50 (*p* = 0.018). At 96 h, although the 1% oxygen group displayed higher metabolic activity (291.12 ± 29.85) than the 5% oxygen group (269.00 ± 26.58), this difference was not statistically significant (*p* = 0.140) (Figure 1C).

These findings suggest that hypoxic conditions (1% O_2_) may transiently suppress metabolic activity at 72 h but allow recovery at later time points (96 h). The absence of significant differences at 48 and 96 h indicates that the metabolic effects of hypoxia may be time-dependent and reversible.

### 3.2. Mitotracker Staining

Mitochondrial staining results showed no statistically significant difference (*p* > 0.05) between cells cultured under 1% and 5% oxygen conditions at the end of 72 h. Confocal microscopy analysis with MitoTracker Green revealed comparable mitochondrial activity in both oxygen concentrations (Figure 1A,B).

### 3.3. Hypoxia Inducible Factor (HIF) 1 Alpha Elisa Results

The analysis of HIF-1α levels across different experimental conditions revealed statistically significant differences (*p* < 0.05, ANOVA), indicating that oxygen concentration and incubation time influence HIF-1α expression in mesenchymal stem cells (MSCs) and their secretome.

Post hoc Tukey HSD analysis revealed that significant differences were observed between conditioned medium groups, particularly between 48 h and 72 h incubation at 1% O_2_, where the highest HIF-1α levels were detected in the 72 h, 1% O_2_ group (*p* < 0.05).

Similarly, cell lysate groups exhibited significant reductions in HIF-1α levels under prolonged hypoxic conditions (1% O_2_), particularly at 72 h and 96 h, suggesting a potential adaptation mechanism to low oxygen exposure (*p* < 0.05, Bonferroni correction).

**Figure 2:**
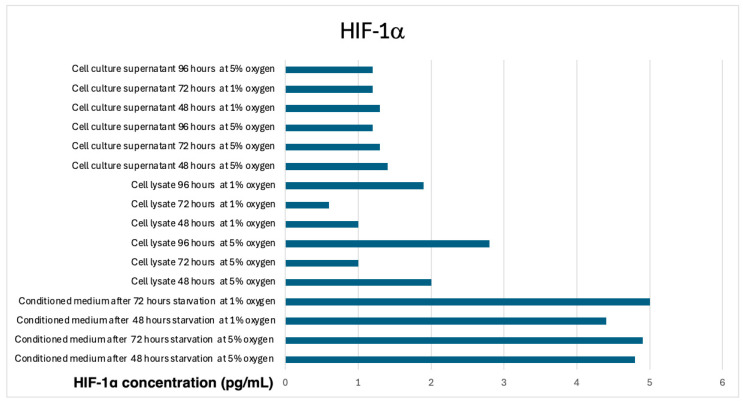
HIF-1α Levels in conditioned media, cell lysates, and cell culture supernatants under different oxygen conditions. The bar graph represents the HIF-1α levels measured by ELISA in conditioned media, cell lysates, and cell culture supernatants of mesenchymal stem cells (MSCs) cultured under different oxygen conditions (5% and 1% O_2_) and starvation durations (48, 72, and 96 h).

These findings indicate that hypoxic conditions (1% O_2_) induce a time-dependent increase in HIF-1α levels in the secretome (conditioned medium), while intracellular HIF-1α levels in cell lysates decrease over time, potentially due to hypoxia-induced degradation or reduced transcriptional activation in prolonged culture (Figure 2).

### 3.4. Conditioned Media Tests

#### 3.4.1. Total Protein Elisa Results

The total protein concentration was significantly affected by oxygen concentration and starvation duration (*p* < 0.001). ANOVA results demonstrated a strong effect size (η^2^ = 0.906), indicating a substantial impact of culture conditions on protein secretion.

**Figure 3:**
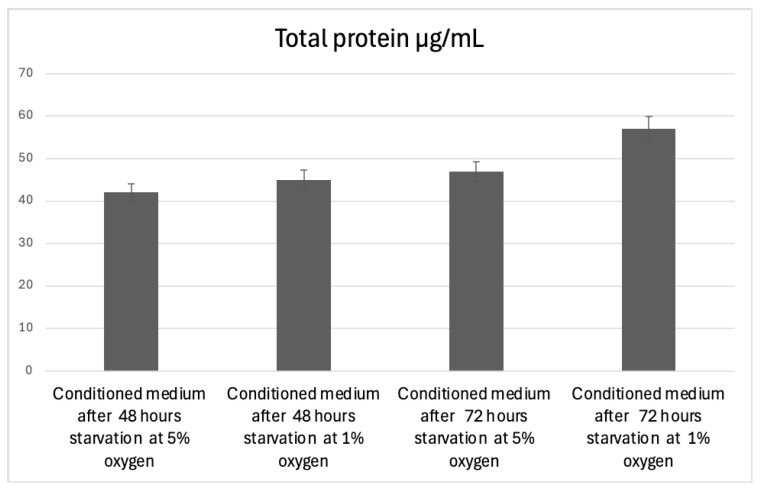
Total protein concentration in conditioned media under different oxygen and starvation conditions. The bar graph illustrates the total protein concentration (µg/mL) in conditioned media collected from mesenchymal stem cells (MSCs) exposed to different oxygen levels (5% and 1% O_2_) and starvation durations (48 and 72 h).

Post hoc analysis revealed that Group 4 (72 h, 1% O_2_) had significantly higher total protein levels compared to all other groups (*p* < 0.001). However, no significant differences were observed between 48 h groups (5% vs. 1% O_2_) or between 48 h and 72 h groups under 5% O_2_ (*p* > 0.05) (Figure 3). These findings suggest that prolonged hypoxia (1% O_2_) enhances total protein secretion in conditioned media, particularly at 72 h.

#### 3.4.2. DLS Results

The Z-average particle size values significantly differed among groups (*p* < 0.05, ANOVA). Post hoc analysis (Tukey HSD) confirmed that all pairwise comparisons were statistically significant, indicating that both oxygen concentration and starvation duration had a substantial effect on the size distribution of particles in the conditioned medium.

At 48 h, the 1% oxygen group exhibited significantly larger particle sizes (300 ± 5 nm) compared to the 5% oxygen group (198 ± 2 nm, *p* < 0.05), suggesting enhanced aggregation or vesicle formation under hypoxic conditions.

At 72 h, the 5% oxygen group had an average particle size of 263 ± 3 nm, whereas the 1% oxygen group showed a broader size distribution, with values ranging from 150 to 245 nm. The reduction in particle size in the 1% oxygen, 72 h group was statistically significant compared to all other groups (*p* < 0.05).

**Figure 4:**
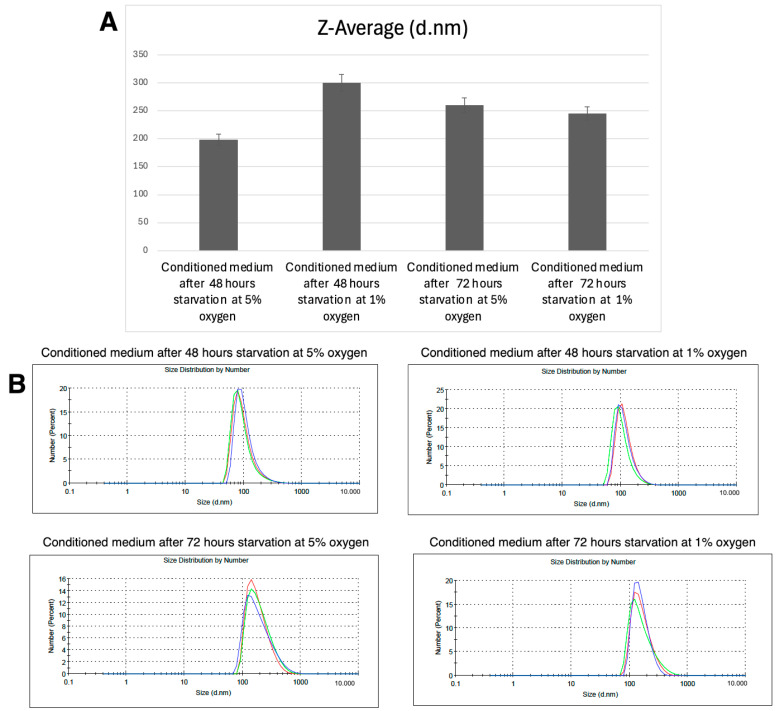
Nanoparticle Size Distribution in conditioned media under different oxygen conditions: (**A**) The bar graph represents the Z-average (d.nm) values of nanoparticles detected in the conditioned media of mesenchymal stem cells (MSCs) cultured under different oxygen conditions (5% and 1% O_2_) and starvation durations (48 and 72 h). (**B**) Dynamic light scattering (DLS) analysis shows the size distribution of nanoparticles in the conditioned media at different experimental conditions. Each graph represents the distribution profile of nanoparticles detected under 5% and 1% oxygen conditions at 48 and 72 h of starvation. The blue, green, and red lines represent three independent measurements.

These results suggest that severe 1% oxygen initially promotes particle aggregation (48 h) but leads to a decrease in average particle size at 72 h, whereas 5% oxygen results in more stable and consistent particle sizes over time (Figure 4).

#### 3.4.3. Zeta Potential Results

**Figure 5:**
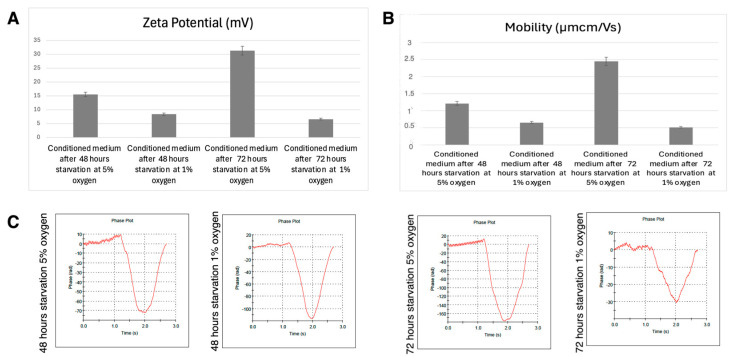
Zeta potential, electrophoretic mobility, and Phase Plot Analysis of conditioned media. (**A**) Zeta Potential (mV): The bar graph represents the zeta potential values of conditioned media collected from mesenchymal stem cells (MSCs) subjected to different oxygen (5% and 1%) and starvation (48 and 72 h) conditions. The results indicate that conditioned media obtained from 72 h starvation at 5% oxygen exhibited the highest zeta potential, suggesting improved colloidal stability and potential changes in extracellular vesicle surface charge under these conditions. (**B**) Electrophoretic Mobility (µmcm/Vs): The mobility values of nanoparticles in conditioned media were significantly influenced by oxygen concentration and starvation duration. The highest mobility was observed in the conditioned media collected after 72 h of starvation at 5% oxygen, indicating increased surface charge and stability, whereas 1% oxygen exposure resulted in lower mobility values, suggesting altered vesicle composition or aggregation behavior. (**C**) Phase Plot Analysis: Phase plots illustrate the dynamic behavior of nanoparticles in conditioned media under different experimental conditions. The data suggest that prolonged hypoxic exposure (1% oxygen) influences nanoparticle phase characteristics, potentially affecting their stability and bioactivity.

The zeta potential values significantly differed among groups (*p* < 0.05, ANOVA). Post hoc analysis (Tukey HSD) confirmed that all pairwise comparisons were statistically significant, indicating that both oxygen concentration and starvation duration had a considerable effect on the surface charge of conditioned media (Figure 5A,C).

At 48 h, the 5% oxygen group exhibited a significantly lower zeta potential (−15.5 ± 0.2 mV) compared to the 1% oxygen group (−8.35 ± 0.02 mV, *p* < 0.05), suggesting a stronger electrostatic repulsion under normoxic conditions.

At 72 h, the 5% oxygen group displayed the lowest zeta potential (−31.3 ± 0.2 mV), which was significantly different from the 1% oxygen group (−6.57 ± 0.02 mV, *p* < 0.05).

The data indicate that hypoxic conditions (1% oxygen) led to a less negative zeta potential, while normoxic conditions (5% oxygen) under prolonged starvation (72 h) resulted in the most negative zeta potential, suggesting enhanced stability in the conditioned medium.

#### 3.4.4. Mobility Results

The electrophoretic mobility values significantly differed among groups (*p* < 0.05, ANOVA). Post hoc analysis (Tukey HSD) confirmed that all pairwise comparisons were statistically significant, indicating that both oxygen concentration and starvation duration had a notable effect on particle mobility in the conditioned medium.

At 48 h, the 5% oxygen group exhibited significantly lower mobility (−1.212 ± 0.002 μmcm/Vs) compared to the 1% oxygen group (−0.654 ± 0.002 μmcm/Vs, *p* < 0.05), suggesting altered surface charge properties under hypoxic conditions (Figure 5B).

At 72 h, the 5% oxygen group displayed the most negative mobility values (−2.451 ± 0.002 μmcm/Vs), which were significantly different from the 1% oxygen group (−0.514 ± 0.002 μmcm/Vs, *p* < 0.05).

The data suggest that longer starvation (72 h) under normoxic conditions (5% oxygen) led to the highest absolute electrophoretic mobility, whereas hypoxic conditions (1% oxygen) resulted in significantly lower mobility, potentially influencing stability and aggregation behavior in the conditioned medium.

### 3.5. Oxidative Stress Analysis Results

#### 3.5.1. Total Antioxidant Status (TAS) Results

**Figure 6:**
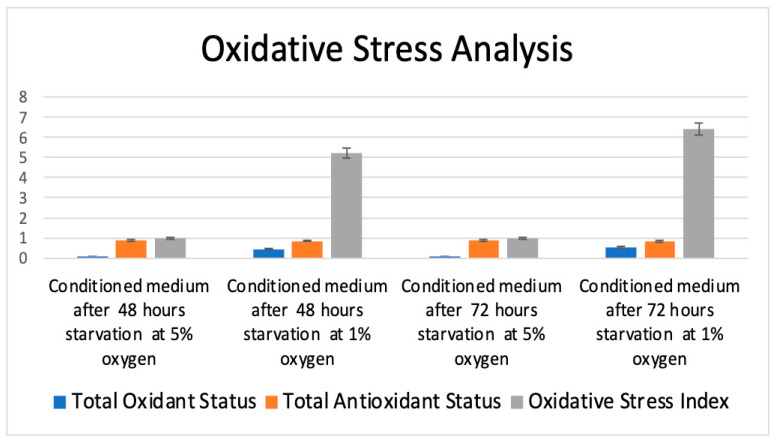
Oxidative stress analysis of conditioned media. This figure presents the oxidative stress analysis of conditioned media derived from mesenchymal stem cells (MSCs) exposed to different oxygen conditions (5% and 1%) and starvation durations (48 and 72 h). The bar graph illustrates the total oxidant status (TOS, blue), total antioxidant status (TAS, orange), and oxidative stress index (OSI, green) for each experimental condition. The results indicate that conditioned media obtained from MSCs exposed to 1% oxygen exhibited significantly higher oxidative stress levels, particularly after 72 h of starvation, as evidenced by the elevated OSI values. In contrast, cells cultured under 5% oxygen showed a more balanced oxidative environment, with lower OSI values and stable TAS levels.

The evaluation of total antioxidant status (TAS) revealed statistically significant differences among the experimental groups (*p* < 0.05, ANOVA). Post hoc comparisons using the Bonferroni correction demonstrated significant differences between the groups cultured under 48 h of starvation at 5% oxygen and 48 h of starvation at 1% oxygen, as well as between the groups cultured under 48 h of starvation at 5% oxygen and 72 h of starvation at 1% oxygen. Additionally, a significant difference was observed between the groups cultured under 72 h of starvation at 5% oxygen and 72 h of starvation at 1% oxygen (*p* < 0.001 for all comparisons). However, no statistically significant differences were observed between the groups cultured under 48 h of starvation at 5% oxygen and 72 h of starvation at 5% oxygen (*p* = 0.141), between the groups cultured under 48 h of starvation at 1% oxygen and 72 h of starvation at 5% oxygen (*p* = 0.013), or between the groups cultured under 48 h of starvation at 1% oxygen and 72 h of starvation at 1% oxygen (*p* = 0.019) (Figure 6).

#### 3.5.2. Total Oxidant Status (TOS) Results

The evaluation of total oxidant status (TOS) revealed statistically significant differences among the experimental groups (*p* < 0.05, ANOVA). Post hoc comparisons using the Bonferroni correction indicated that all pairwise comparisons were statistically significant, except between the groups subjected to 48 h of starvation at 5% oxygen and 72 h of starvation at 5% oxygen.

No statistically significant difference was observed between the groups subjected to 48 h of starvation at 5% oxygen and 72 h of starvation at 5% oxygen (*p* = 0.995), while all other intergroup comparisons demonstrated significant differences (*p* < 0.001, Bonferroni post hoc test) (Figure 6).

#### 3.5.3. Oxidative Stress Index

The analysis of the oxidative stress index (OSI) revealed statistically significant differences among the experimental groups (*p* < 0.05, ANOVA). Post hoc comparisons using the Bonferroni correction indicated that all pairwise comparisons were statistically significant (*p* < 0.001), except between the groups exposed to 48 h of starvation at 5% oxygen and 72 h of starvation at 5% oxygen, where no significant difference was observed (*p* = 1.00) (Figure 6).

These results indicate that exposure to 1% oxygen significantly increases oxidative stress in mesenchymal stem cell-derived conditioned media, particularly after 72 h of starvation, as evidenced by the substantially higher OSI values in the 1% oxygen groups compared to those cultured at 5% oxygen. The absence of significant differences between the 48 h and 72 h starvation groups under 5% oxygen suggests that oxygen concentration, rather than starvation duration, is the primary determinant of oxidative stress levels in MSC secretoms.

## 4. Discussion

This study investigated the effects of hypoxic preconditioning at 1% and 5% oxygen on mesenchymal stem cell (MSC)-derived conditioned media, focusing on oxidative stress markers, metabolic activity, and physicochemical properties. The results demonstrated that hypoxia significantly influenced oxidative stress levels and the biochemical composition of the conditioned media, with prolonged exposure to 1% oxygen leading to increased oxidative stress indices and altered nanoparticle characteristics.

The metabolic activity of MSCs, as assessed by the MTT assay, showed a significant reduction in cells cultured under 1% oxygen at 72 h compared to those maintained at 5% oxygen. However, no significant differences were observed at 48 and 96 h, indicating that hypoxia-induced metabolic suppression may be transient and subject to adaptation over time. This aligns with previous findings suggesting that hypoxic conditions initially reduce mitochondrial respiration but promote glycolytic adaptations, allowing cells to maintain energy production under low oxygen availability [25,26].

An analysis of oxidative stress markers revealed that total oxidant status (TOS) was significantly elevated in conditioned media derived from MSCs exposed to 1% oxygen, particularly after 72 h of starvation. The oxidative stress index (OSI) further confirmed that hypoxia imposed a substantial oxidative burden on the cells, with the highest OSI values recorded in the 72 h, 1% oxygen group. These findings are consistent with previous research indicating that hypoxia enhances the generation of reactive oxygen species (ROS) in MSC cultures, leading to a shift in redox homeostasis and potential modifications in the secretory profile of the cells [27,28]. The reduction in total antioxidant status (TAS) under 1% oxygen further supports the notion that hypoxia alters the balance between oxidant and antioxidant mechanisms, potentially affecting the therapeutic efficacy of MSC-derived secretomes.

Hypoxic preconditioning also significantly affected the physicochemical properties of extracellular vesicles (EVs) within the conditioned media. Dynamic light scattering (DLS) analysis showed that nanoparticles secreted under 1% oxygen were larger at 48 h compared to those derived from 5% oxygen cultures, suggesting enhanced aggregation. However, at 72 h, the 1% oxygen group exhibited a broader distribution of particle sizes, with a shift toward smaller vesicles. These findings suggest that hypoxia influences EV biogenesis and stability over time, potentially affecting their therapeutic potential. The observed changes in zeta potential and electrophoretic mobility further indicate that hypoxic conditioning alters the surface charge and colloidal stability of secreted nanoparticles, which may influence their cellular uptake and biodistribution.

The impact of hypoxia on hypoxia-inducible factor 1-alpha (HIF-1α) expression was also evaluated, revealing significant differences between conditioned media, cell lysates, and cell culture supernatants. HIF-1α levels were highest in the conditioned media of MSCs cultured under 1% oxygen at 72 h, supporting previous reports that hypoxia enhances the secretion of bioactive molecules through HIF-1α-mediated pathways [29,30]. However, intracellular HIF-1α levels in cell lysates decreased over time, suggesting a potential adaptation mechanism that limits prolonged HIF-1α activation under chronic hypoxia.

One limitation of this study is the absence of a normoxic control group (21% O_2_), which may restrict the broader interpretation of oxygen-mediated effects. However, it is important to note that mesenchymal stem cells (MSCs), particularly those derived from Wharton’s jelly, naturally reside in physiologically hypoxic environments, with oxygen levels typically ranging from 1% to 7% in vivo [31,32]. As such, the current study intentionally focused on comparing two physiologically relevant oxygen tensions (1% and 5%) to evaluate the differential effects of mild versus severe hypoxia on secretome characteristics. Although a normoxic group (21% O_2_) may offer additional insight into oxidative stress responses under artificially elevated oxygen conditions, its omission in this study was based on the aim to mimic in vivo physiological conditions rather than supraphysiological environments. Future investigations, including a normoxic control, may further elucidate the extent of hypoxia-induced shifts in MSC metabolism and secretory behavior.

The present study was designed to investigate how different hypoxic conditions influence the oxidative stress balance and physicochemical properties of MSC-derived conditioned media. It was not designed to perform a comprehensive proteomic or growth factor profile. In support of hypoxia-induced signaling, we quantified HIF-1α levels, which are known to regulate the transcription of multiple angiogenic and immunomodulatory factors [16,18,30]. Although ELISA-based analysis of key secreted proteins was beyond the scope of this initial investigation, future studies will be directed toward proteomic and transcriptomic profiling to further clarify how oxygen tension modulates the therapeutic cargo of MSC-derived secretomes.

Overall, these findings highlight the complex interplay between oxygen concentration, oxidative stress, and the secretory profile of MSC-derived conditioned media. The results suggest that while hypoxic preconditioning enhances certain regenerative properties, it also imposes oxidative stress, which may influence the stability and functionality of secreted biomolecules. A major limitation of this study is the lack of proteomic and lipidomic analyses of the conditioned media, which would provide a more comprehensive understanding of how hypoxia influences the molecular composition of secreted factors. Future studies should employ mass spectrometry-based proteomic approaches to characterize the protein cargo of EVs derived from hypoxia-preconditioned MSCs.

## 5. Conclusions

This study highlights the impact of oxygen concentration on MSC metabolism, oxidative stress balance, and the physicochemical properties of conditioned media. The findings suggest that prolonged exposure to 1% oxygen enhances antioxidant capacity but also alters extracellular vesicle properties, which may have important implications for regenerative medicine applications. Future research should focus on optimizing hypoxic preconditioning strategies to maximize the therapeutic potential of MSC-derived secretomes. Finally, translating these findings into clinical applications requires a deeper understanding of how hypoxia-induced modifications in MSC-derived EVs affect their biodistribution, immunomodulatory functions, and therapeutic potential in disease models.

## Data Availability

Data are available on request.
